# HDAC6 Regulates Epidermal Growth Factor Receptor (EGFR) Endocytic Trafficking and Degradation in Renal Epithelial Cells

**DOI:** 10.1371/journal.pone.0049418

**Published:** 2012-11-13

**Authors:** Wei Liu, Lucy X. Fan, Xia Zhou, William E. Sweeney, Ellis D. Avner, Xiaogang Li

**Affiliations:** 1 Department of Pediatrics, Medical College of Wisconsin, Milwaukee, Wisconsin, United States of America; 2 Department of Physiology, Medical College of Wisconsin, Milwaukee, Wisconsin, United States of America; 3 Children’s Research Institute, Children’s Hospital of Wisconsin, Milwaukee, Wisconsin, United States of America; 4 Department of Nephrology, Tongji Hospital, Huazhong University of Science and Technology, Wuhan, China; Baylor College of Medicine, United States of America

## Abstract

We present for the first time that histone deacetylase 6 (HDAC6) regulates EGFR degradation and trafficking along microtubules in *Pkd1* mutant renal epithelial cells. HDAC6, the microtubule-associated α-tubulin deacetylase, demonstrates increased expression and activity in *Pkd1* mutant mouse embryonic kidney cells. Targeting HDAC6 with a general HDAC inhibitor, trichostatin (TSA), or a specific HDAC6 inhibitor, tubacin, increased the acetylation of α-tubulin and downregulated the expression of EGFR in *Pkd1* mutant renal epithelial cells. HDAC6 was co-localized with EGF induced endocytic EGFR and endosomes, respectively. Inhibition of the activity of HDAC6 accelerated the trafficking of EGFR from early endosomes to late endosomes along the microtubules. Without EGF stimulation EGFR was randomly distributed while after stimulation with EGF for 30 min, EGFR was accumulated around α-tubulin labeled microtubule bundles. These data suggested that the *Pkd1* mutation induced upregulation of HDAC6 might act to slow the trafficking of EGFR from early endosomes to late endosomes along the microtubules for degradation through deacetylating α-tubulin. In addition, inhibition of HDAC activity decreased the phosphorylation of ERK1/2, the downstream target of EGFR axis, and normalized EGFR localization from apical to basolateral in *Pkd1* knockout mouse kidneys. Thus, targeting HDAC6 to downregulate EGFR activity may provide a potential therapeutic approach to treat polycystic kidney disease.

## Introduction

Autosomal dominant polycystic kidney disease (ADPKD) is a genetic disease caused by mutations in either *PKD1* or *PKD2* and is characterized by the formation and progressive growth of cystic lesions that ultimately destroy normal renal parenchyma.[1], [2], [3] Cyst growth is the result of sustained proliferation of incomplete or de-differentiated epithelial cells and accumulation of fluid within the cysts.[3].

ErbB receptor tyrosine kinases (EGF receptor or EGFR, ErbB2, ErbB3, and ErbB4) and their ligands play important roles in renal development, in renal electrolyte homeostasis and tubule repair following injury.[4], [5], [6], [7], [8], [9], [10] EGFR is normally sorted to basolateral membranes in mature tubular epithelial cells.[8] However, numerous primary PKD causing mutations alter EGFR polarity, leading to increased apical expression and activity.[11] Cystic epithelial cells from ADPKD patients are unusually susceptible to the proliferative stimulus of EGF. EGF and EGF-like ligands are secreted into the apical medium of cultured cystic epithelial cells and are present in cyst fluid from ADPKD patients.[12], [13] Thus, in cystic epithelia, both receptors (ErbB1 and ErbB2) and ligands are expressed on the same side of the cell leading to sustained mitogenic signaling. In addition, increased expression of ErbB2 leads to the formation of ErbB1/ErbB2 heterodimers that also slows internalization and receptor degradation.[14].

Inhibition of EGFR tyrosine kinase activity either genetically or pharmacologically significantly reduces renal cyst formation and improves renal function in rodent models of PKD.[7], [11], [15] These observations suggest that persistent EGF signaling from the apical cell surface of renal epithelia is a primary disease progression factor in PKD. However, the mechanism(s) involved in EGF mediated EGFR stability and endocytic trafficking in cystic epithelial cells is unknown.

Ligand activated EGFR complexes on the apical cell surface are internalized into apical sorting endosomes (ASE) and apical recycling endosomal (AREs) intermediates and trafficked through a series of endocytic compartments where they are either recycled or sorted for proteolytic degradation in the lysosome.[16] Aberrant regulations of these complex sorting pathways have been linked to the development and progression of PKD.[14].

Microtubules, together with the microtubule-based motor proteins, kinesin and cytoplasmic dynein, are involved in sorting and transport of early endocytic vesicles to later stage endocytic compartments.[17], [18] Recent studies suggest that acetylation of α-tubulin, the component of microtubules, affects the stability of microtubule, which further regulates intracellular cargo transport.[17], [18], [19] Histone deacetylase 6 (HDAC6) is associated with the microtubule network and has been shown to regulate intracellular transport of EGFR containing vesicles in some cell types through its tubulin deacetylase activity.[16], [20] In HDAC6-deficient cells, the entire microtubule network becomes hyperacetylated.[19], [21], [22] Whether HDAC6 regulates EGFR endocytic trafficking and degradation through the microtubule mediated vesicular network in cystic epithelial cells is the subject of this study.

In this study, we present for the first time evidence to support the theory that HDAC6 regulates EGFR endocytic trafficking and degradation through modulation of tubulin acetylation in cystic renal epithelial cells. We found that the expression and activity of HDAC6 was upregulated in *Pkd1* mutant renal epithelial cells. We further found that the stability of microtubules affected the reactive pattern of EGFR level after EGF stimulation in *Pkd1* mutant kidney epithelial cells. Furthermore, HDAC6 inhibition leads to the stimulation of microtubule-dependent transport of EGFR containing vesicles and the degradation of EGFR, and normalizes EGFR localization from apical to basolateral.

## Results

### HDAC6 Expression is Increased in *Pkd1* Mutant Kidney Epithelial Cells

**Figure 1:**
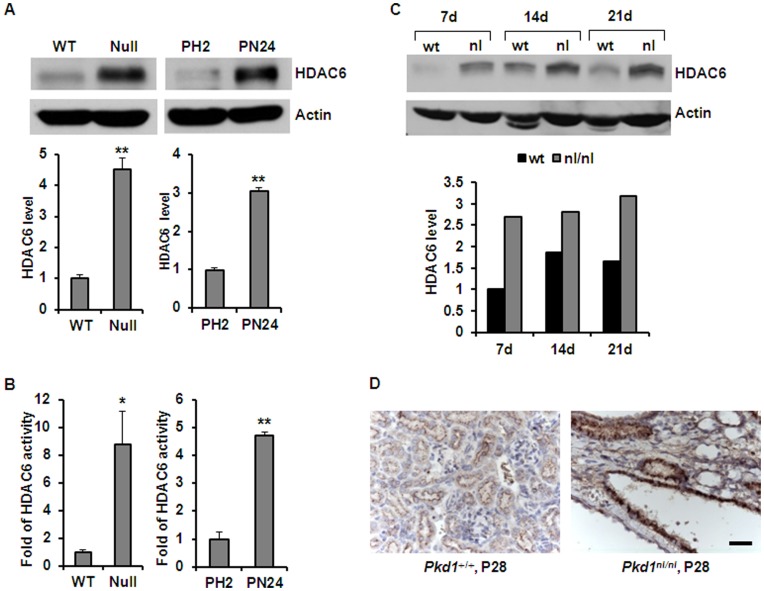
HDAC6 was up-regulated in *Pkd1* mutant renal epithelial cells and kidney tissues. (A) Western blotting analysis of the expression of HDAC6 in *Pkd1* wild-type (WT) and *Pkd1^null/null^* (Null) MEK cells as well as in postnatal *Pkd1* heterozygous PH2 cells and homozygous PN24 cells. The expression of HDAC6 in the above cells was quantified from three independent immunoblots and was standardized to actin. ** *p < 0.01*. (B) The activity of HDAC6 in *Pkd1* wild-type (WT) and *Pkd1^null/null^* (Null) MEK cells as well as in postnatal *Pkd1* heterozygous PH2 cells and homozygous PN24 cells. The activity of HDAC6 was increased about 7.8 fold in *Pkd1^null/null^* (Null) MEK cells and 4.8 fold in postnatal *Pkd1* homozygous PN24 cells compared with that in *Pkd1* wild type MEK cells and postnatal *Pkd1* heterozygous PH2 cells, respectively. The HDAC6 activity assay was performed in three independent experiments. * *p < 0.05*, ** *p < 0.01*. (C) Western blotting analysis of the expression of HDAC6 in *Pkd1* wild type and *Pkd1* hypomorphic homozygous (*Pkd1^nl/nl^*) kidney tissues collected at postnatal days 7, 14, and 21. The relative expression levels of HDAC6 in different kidneys were standardized to actin and were presented in the lower panel. (D) Immunohistochemistry of HDAC6 in kidney sections from *Pkd1* wild type and *Pkd1^nl/nl^* mice harvested at postnatal day 28. Scale bar, 20 µm.

Recent evidence suggest that sorting and transport of early endocytic cargo to later stage endocytic compartments require microtubules, and HDAC6 may be involved in this process by regulating the stability of microtubules through deacetylating α-tubulin.[16], [20] We found that HDAC6 was up-regulated in DBA (dolichos biflorus agglutinin) positive *Pkd1* mutant mouse embryonic kidney (MEK) epithelial cells compared to age-matched *Pkd1* wild type MEK cells. HDAC6 expression was also increased in proximal tubule cells derived from postnatal day 24 (PN24) *Pkd1* homozygous (Null) kidneys compared to proximal tubule cells derived from postnatal *Pkd1* heterozygous kidneys (PH2) (Fig. 1A). In addition, we found that HDAC6 activity was significantly increased in *Pkd1* mutant cystic epithelial cells compared with *Pkd1* wild type and heterozygous control cells (Fig. 1B). HDAC6 expression was also increased in kidney tissues from well characterized, hypomorphic homozygous *Pkd1^nl/nl^* mice [23] harvested at postnatal days 7, 14 and 21, when compared to that in age matched *Pkd1* wild type mouse kidneys (Fig. 1C). Our immunohistochemistry results further supported that HDAC6 was upregulated in cyst lining epithelia of *Pkd1* ^nl/nl^ mouse kidneys harvest at postnatal day 28 compared to that in the normal renal tubules of the age matched *Pkd1* wild type mouse kidneys and the upregulated HDAC6 was localized in the cytosol, the site where HDAC6 was found in all the other cell types reported, of cystic epithelial cells (Fig. 1D). These results demonstrate an inverse relationship between PC1 expression and HDAC6 expression levels in renal epithelial cells.

### Inhibition of HDAC6 Activity with Tubacin, a Specific HDAC6 Inhibitor, or TSA, a Pan HDAC Inhibitor, Decreased the Expression of EGFR in Renal Epithelial Cells

**Figure 2:**
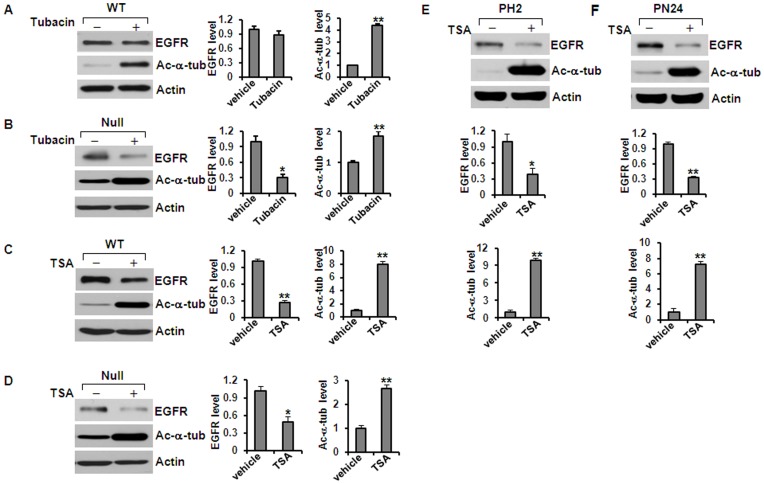
Inhibition of HDAC6 activity decreased the levels of EGFR and increased acetyl-α-tubulin in renal epithelial cells. (A) and (B) Western blotting analysis of the expression of EGFR and acetyl-α-tubulin in *Pkd1* wild-type (WT) MEK cells (A) and *Pkd1^null/null^* (Null) MEK cells (B) treated with or without tubacin. Tubacin (5 µM) treatment deceased the levels of EGFR in *Pkd1* mutant MEK cells, but increased the acetyl-α-tubulin in both wild-type and mutant MEK cells. (C) and (D) Western blotting analysis of the expression of EGFR and acetyl-α-tubulin in *Pkd1* wild-type (WT) MEK cells (C) and *Pkd1^null/null^* (Null) MEK cells (D) treated with or without TSA (100 ng/ml). TSA treatment decreased the levels of EGFR and increased the acetyl-α-tubulin in *Pkd1^null/null^* (Null) MEK cells. (E) and (F) TSA (200 ng/ml) has the same effect on the expression of EGFR and acetyl-α-tubulin in postnatal *Pkd1* heterozygous PH2 and homozygous PN24 cells. * *p < 0.05*. ** *p < 0.01*.

**Figure 3:**
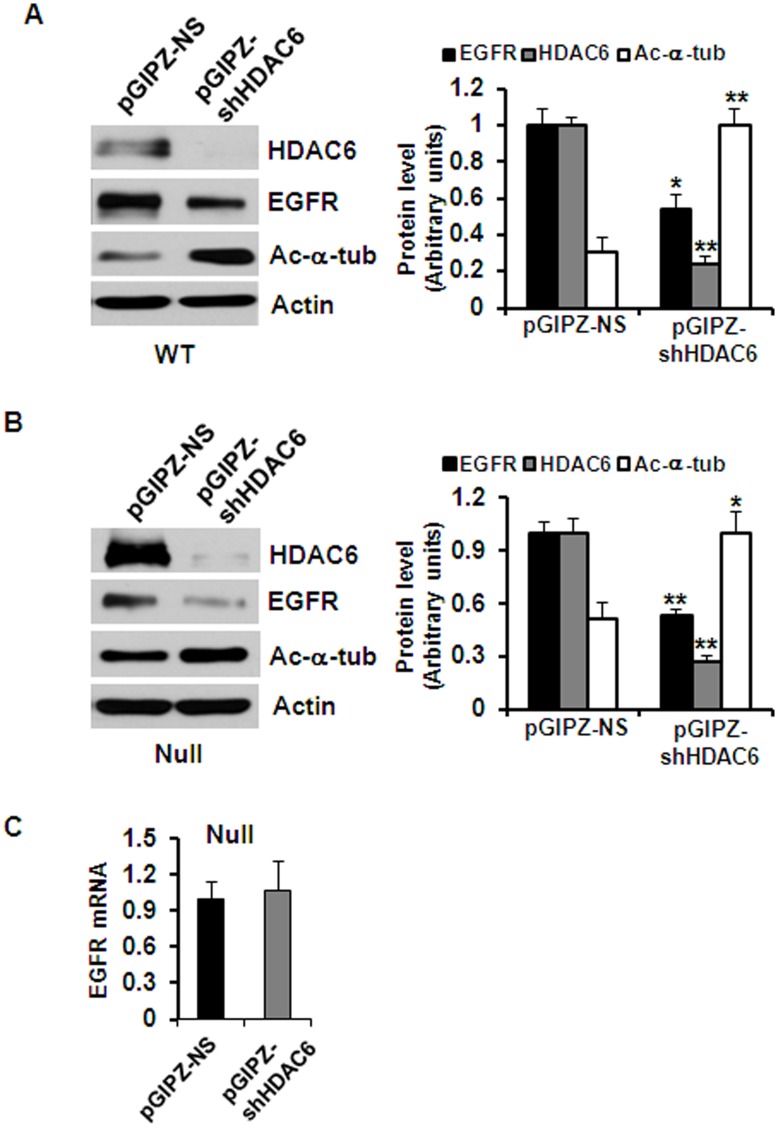
Knockdown HDAC6 with shRNA increases EGFR degradation. (A) and (B) Western blotting analysis of the expression of HDAC6, EGFR and acetyl-α-tubulin in *Pkd1* wild-type (WT) MEK cells (A) and *Pkd1^null/null^* (Null) MEK cells (B) transduced with lentiviral vector pGIPZ-shHDAC6 mediated HDAC6 shRNA or control lentiviral empty vector. The levels of EGFR were reduced and the levels of acetyl-α-tubulin were increased in HDAC6 knockdown cells. (C) EGFR expression at mRNA level in *Pkd1* mutant (Null) MEK cells with HDAC6 shRNA or vector (control) was analyzed by real-time PCR. Knockdown HDAC6 with shRNA did not affect the transcription of EGFR. Average values from triplicated experiments were shown.

EGFR has been found over-expressed and hyper-activated in human ADPKD epithelia and in mouse models of ADPKD.[3] To investigate whether the upregulated HDAC6 affected EGFR levels in kidney epithelial cells, we inhibited HDAC6 activity with either tubacin or TSA. We found that treatment with either tubacin or TSA decreased the levels of EGFR but increased the acetylation of α-tubulin in *Pkd1* wild type and mutant kidney epithelial cells compared to untreated control cells (Fig. 2A–F). It is interesting to note that TSA treatment decreased the levels of EGFR in both *Pkd1* wild type and mutant MEK cells while tubacin treatment only decease the levels of EGFR in *Pkd1* mutant MEK cells (Fig. 2A and 2B). In addition, we found that TSA treatment significantly increased the acetyl-α-tubulin staining in *Pkd1* wild type and mutant MEK cells as well as in PH2 and PN24 cells (data not show). Furthermore, knockdown of HDAC6 with pGIPZ lentiviral vector mediated HDAC6 shRNA in both *Pkd1* wild type and mutant MEK cells decreased the expression of EGFR and increased the acetylation of α-tubulin in MEK cells compared to the control MEK cells transduced with the empty vector (Fig. 3A and 3B). In addition, we found that knockdown of HDAC6 with shRNA did not reduce EGFR expression at mRNA level in *Pkd1* null cells (Fig. 3C). These results suggested that HDAC6 might regulate the degradation of EGFR through deacetylation of α-tubulin.

### HDAC6 Associated with the Endosomal Compartments in Kidney Epithelial Cells

**Figure 4:**
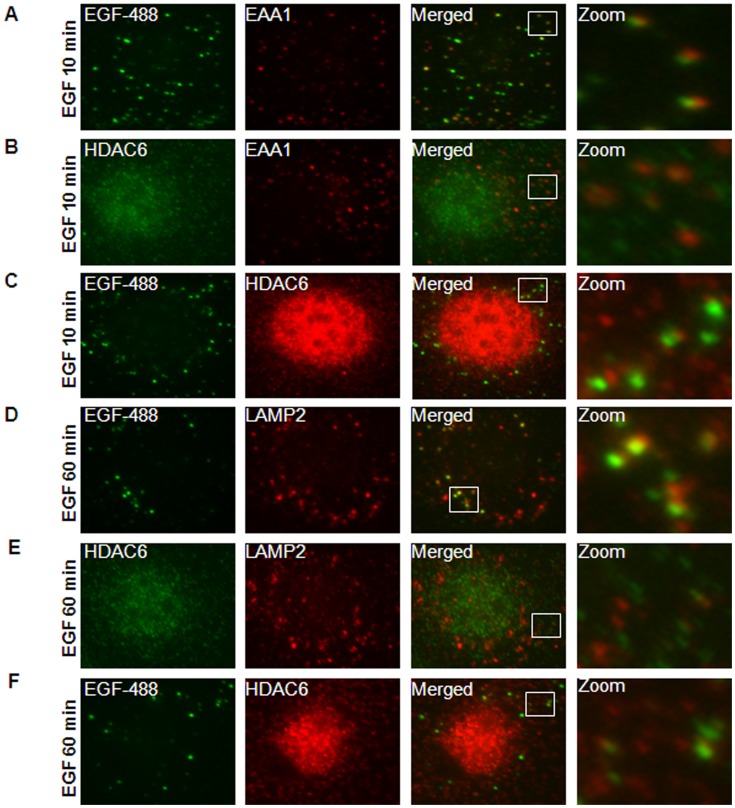
HDAC6 associated with the endosomal compartments in mIMCD3 cells. (A) mIMCD3 cells were serum-starved overnight and then treated with 80 ng/ml EGF-Alexa Fluor 488 for 10 min. After that, cells treated with 0.005% saponin were stained with anti EEA1 antibody. Image in *zoom* shows the example of early endosomes stained with EEA1 (*red*) colocalized with EGFR (*green*). (B) Serum-starved mIMCD3 cells were treated with 100 ng/ml EGF for 10 min and then stained with anti-HDAC6 and EEA1 antibodies. Image in *zoom* shows the example of early endosomes stained with EEA1 (*red*) colocalized with HDAC6 (*green*). (C) mIMCD3 cells were treated and processed as in (A) except using anti-HDAC6 antibody. Image in *zoom* shows the colocalization of EGFR (*green*) with HDAC6 (red). (D) mIMCD cells were treated with 80 ng/ml EGF-Alexa Fluor 488 for 60 min and then stained with anti-LAMP2 antibody. Image in *zoom* shows the colocalization of EGFR (*green*) with later endosomes stained with LAMP2 (*red*). (E) mIMCD3 cells were treated with 100 ng/ml EGF for 60 min and then stained with anti-HDAC6 and anti-LAMP2 antibodies. Image in *zoom* shows the example of later endosomes stained with LAMP2 (*red*) colocalized with HDAC6 (*green*). (F) mIMCD3 cells were treated and processed as in (D) except using anti-HDAC6 antibody. Image in *zoom* shows the colocalization of EGFR (*green*) with HDAC6 (red).

**Figure 5:**
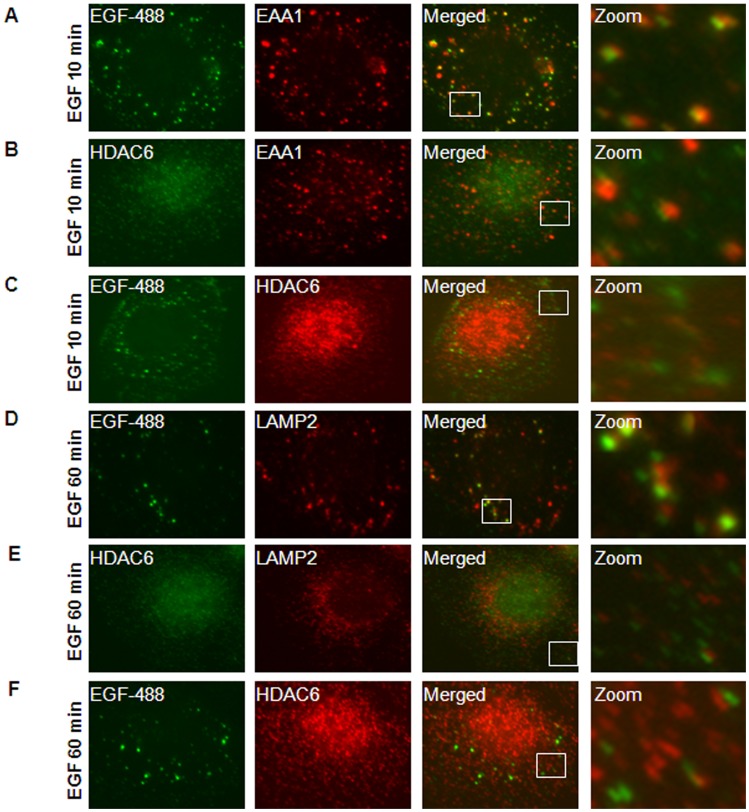
HDAC6 associated with the endosomal compartments in PN24 cells. (A) PN24 cells were serum-starved overnight and treated with 80 ng/ml EGF-Alexa Fluor 488 for 10 min. After that, cells treated with 0.005% saponin were stained with anti EEA1 antibody. Image in *zoom* shows the example of early endosomes stained with EEA1 (*red*) colocalized with EGFR (*green*). (B) Serum-starved PN24 cells were treated with 100 ng/ml EGF for 10 min and then stained with anti-HDAC6 and EEA1 antibodies. Image in *zoom* shows the example of early endosomes stained with EEA1 (*red*) colocalized with HDAC6 (*green*). (C) PN24 cells were treated and processed as in (A) except using anti-HDAC6 antibody. Image in *zoom* shows the colocalization of EGFR (*green*) with HDAC6 (red). (D) PN24 cells were treated with 80 ng/ml EGF-Alexa Fluor 488 for 60 min and then stained with anti-LAMP2 antibody. Image in *zoom* shows the colocalization of EGFR (*green*) with later endosomes stained with LAMP2 (*red*). (E) PN24 cells were treated with 100 ng/ml EGF for 60 min and then stained with anti-HDAC6 and anti-LAMP2 antibodies. Image in *zoom* shows the example of later endosomes stained with LAMP2 (*red*) colocalized with HDAC6 (*green*). (F) PN24 cells were treated and processed as in (D) except using anti-HDAC6 antibody. Image in *zoom* shows the example of the colocalization of EGFR (*green*) with HDAC6 (red).

**Figure 6:**
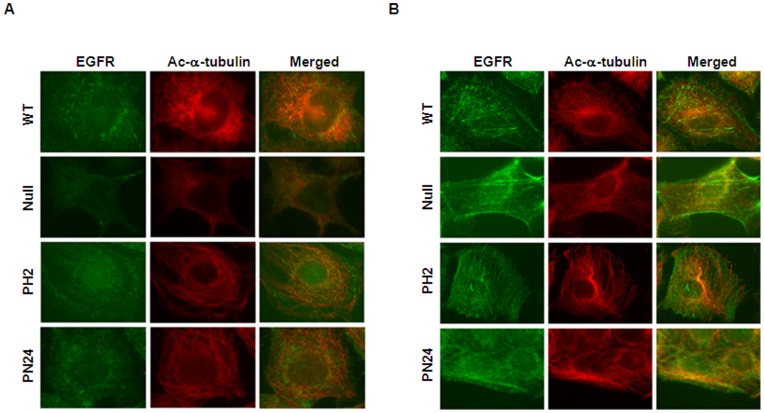
EGFR was accumulated around microtubules following EGF stimulation. (A) EGFR was randomly distributed without EGF stimulation. Four kinds of renal epithelial cells (WT, Null, PH2, PN24) were serum-starved overnight and were then treated with 0.005% saponin before staining with anti-EGFR (*green*) and anti-acetyl-α-tubulin *(red)* antibodies. (B) EGFR was accumulated around microtubules following EGF stimulation. Serum-starved renal epithelial cells were stimulation with EGF for 30 min. The cells were then processed for immunostaining as in (A). EGFR (*green*) was accumulated around microtubule bundles labeled by acetyl-α-tubulin staining (*red*).

To investigate the connection between HDAC6 and EGFR endocytic trafficking in renal epithelial cells, we first examined the association of HDAC6 with endocytic EGFR. Murine inner medullary collecting duct (mIMCD3) cells were stimulated with EGF for 10 minutes, and EGFR labeled with EGF-Alexa Fluor 488 co-localized on the early endosomes labeled with EEA1, an early endosome marker (Fig. 4A). At the same time, HDAC6 also colocalized with a significant portion of early endosomes labeled by EEA1 (Fig. 4B) and EGF-Alexa Fluor 488, respectively (Fig. 4C). These results suggest that HDAC6 interacts with the early endosomal compartment and endocytic EGFR. After cells were stimulated with EGF for 60 minutes, both HDAC6 and EGFR were colocalized with later endosomes marked by LAMP2 (Fig. 4D–4F). Colocalization of HDAC6 with EGFR as well as early and later endosomes was also confirmed in postnatal *Pkd1* homozygous PN24 cells stimulated with EGF for 10 and 60 minutes (Fig. 5A–5F). In addition, we found that without EGF stimulation EGFR was randomly distributed in *Pkd1* wild type and mutant MEK cells as well as in postnatal *Pkd1* heterozygous PH2 and homozygous PN24 cells stained with anti-EGFR antibody (Fig. 6A). However, after stimulation with EGF for 30 min, EGFR accumulated around microtubule bundles in renal epithelial cells as shown by labeled α-tubulin staining (Fig. 6B) and this distribution pattern disappeared with EGF treatment for 60 minutes (data not shown).

### Inhibition of HDAC Activity Increased the Transport of the Activated EGFR from the Early Endosomes to Late Endosomes/Lysosomes

**Figure 7:**
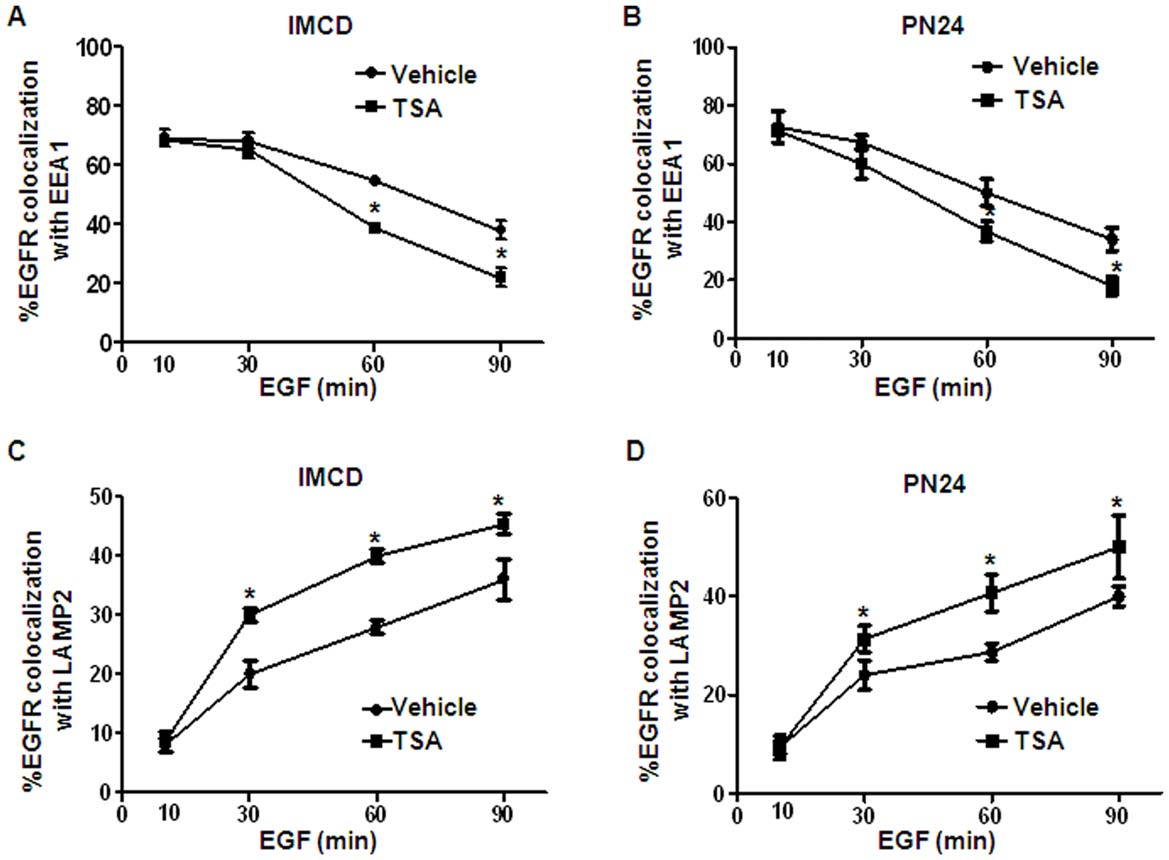
HDAC6 regulates EGFR intracellular transport. (A) mIMCD3 cells were serum-starved and then stimulated with 100 ng/ml EGF for indicated periods of time. The cells were then processed for double-labeling immunostaining with anti-EEA1 and anti-EGFR antibodies. Localization of EGFR to the EEA1-containing vesicles was analyzed. (B) PN24 cells were serum-starved and then stimulated with 100 ng/ml EGF for indicated periods of time. The cells were then processed for immunostaining as in (A). Localization of EGFR to the EEA1-containing vesicles was analyzed. (C) mIMCD3 cells were processed for double-labeling immunostaining with anti-LAMP2 and anti-EGFR antibodies. Co-localization of EGFR to LAMP-2-containing vesicles was examined. (D) PN24 cells were processed for double-labeling immunostaining with anti-LAMP2 and anti-EGFR antibodies. Co-localization of EGFR to LAMP-2-containing vesicles was examined. * *p < 0.05*.

We investigated whether HDAC6 affected EGFR transport from early endosomes to late endosomes/lysosomes by inhibiting HDAC6 activity with TSA. We found that although the degree of colocalization of EGFR with early endosome marker EEA1 decreased with time in mIMCD3 and PN24 cells stimulated with EGF alone (Fig. 7A and 7B), the percentage of vesicles which were positive for both EGFR and EEA1 decreased more quickly in these cells after cotreatment with EGF and TSA (Fig. 7A and 7B). We further found that more EGFR was co-localized with the late endosome and lysosome marker LAMP2 in mIMCD3 and PN24 cells upon co-treatment with EGF and TSA at later time points (Fig. 7C and 7D). These findings suggested that inhibition of HDAC activity accelerated the transport of the activated EGFR from the early endosomes to late endosomes/lysosomes.

### Microtubule Stability Affected EGF Induced EGFR Degradation

**Figure 8:**
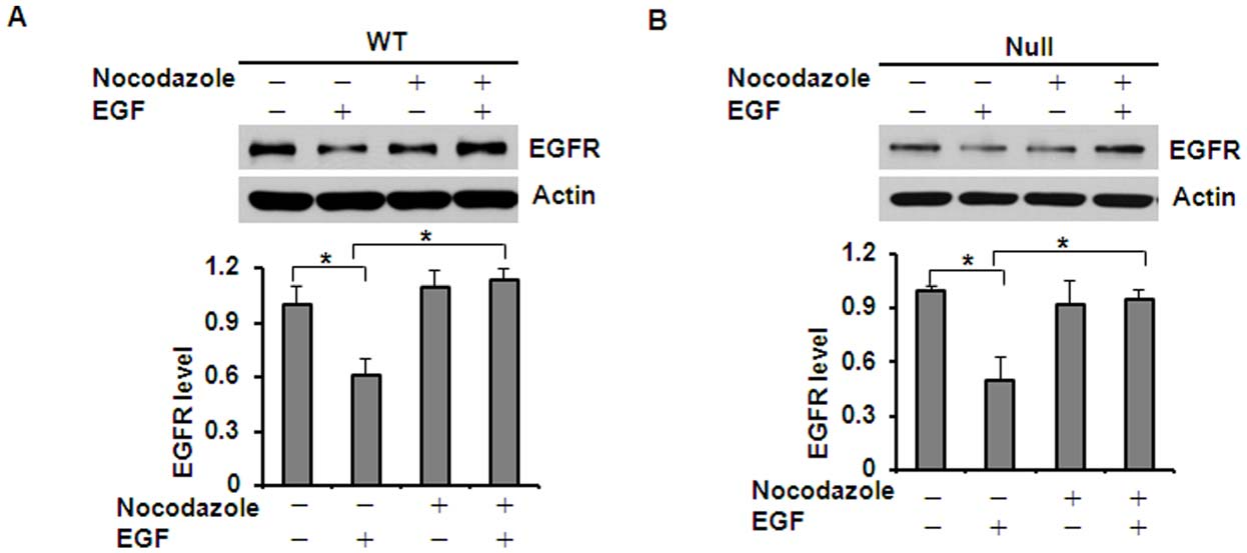
Microtubule stability affected EGF induced EGFR degradation. (A) and (B) Influence of nocodazole on EGF-induced EGFR degradation. *Pkd1* wild-type (WT) MEK cells (A) and *Pkd1^null/null^* (Null) MEK cells (B) were pretreated with 10 µg/ml cycloheximide for 2 hours. Pretreated cells were treated with 0.2 µg/ml nocodazole and/or 100 ng/ml EGF for another 2 hours. EGF-induced EGFR degradation was strikingly attenuated after co-treatment with EGF and nocodazole, which disturbed the stability of microtubule. * *p < 0.05*. ** *p < 0.01*.

**Figure 9:**
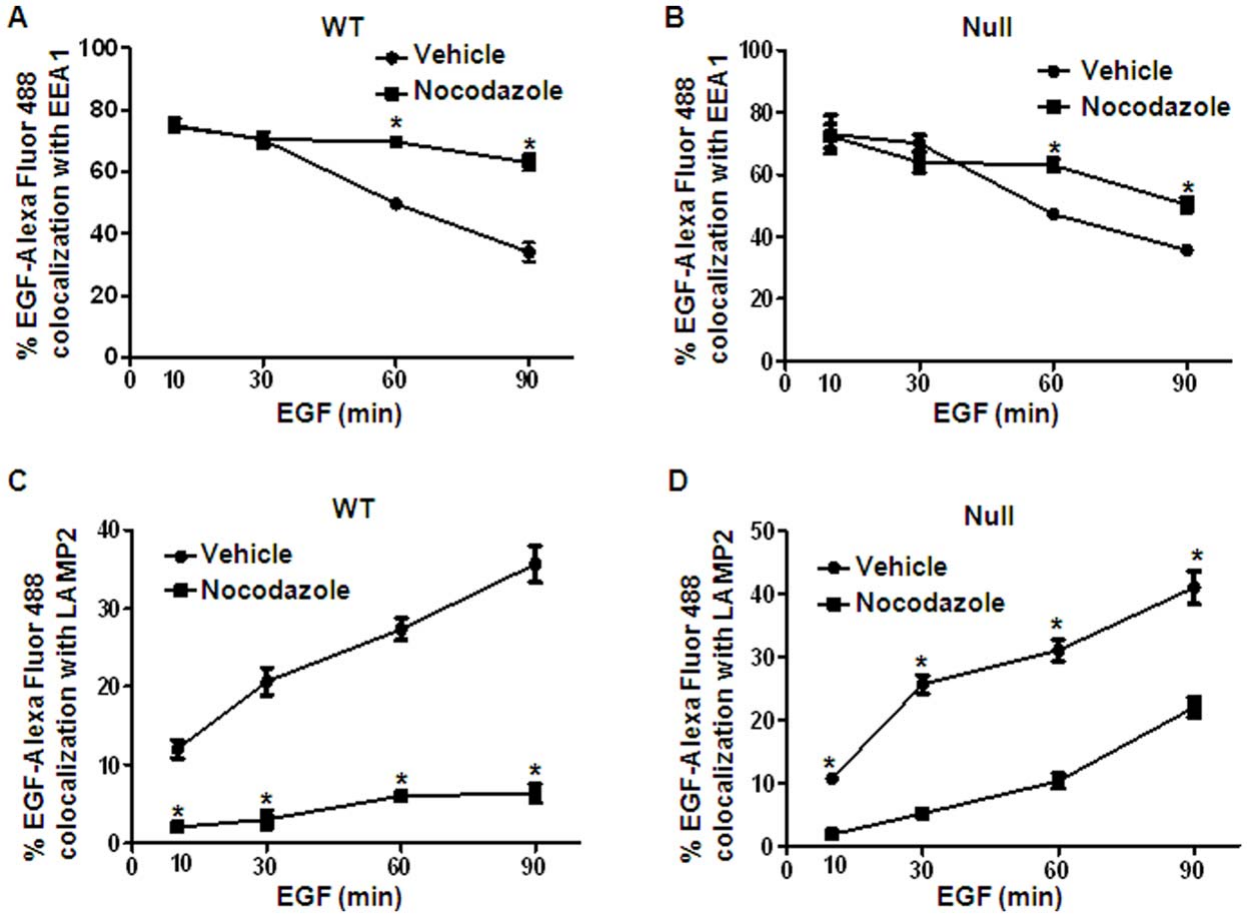
Treatment with nocodazole decreased EGFR intracellular transport. (A) *Pkd1* wild type MEK cells were serum-starved and preteated with nocodazole (0.2 µg/ml) for 30 min. The pretreated cells were further treated with nocodazole and 80 ng/ml EGF-Alexa Fluor 488 for indicated periods of time. Then, cells were stained with anti-EEA1 antibody. Localization of EGF-Alexa Fluor 488 to the EEA1-containing vesicles was analyzed. * *p < 0.05*. (B) *Pkd1* mutant (Null) MEK cells were treated, processed for immunostaining, and analyzed as in (A). * *p < 0.05*. (C) *Pkd1* wild type MEK cells were treated as in (A) and then processed for immunostaining with anti-LAMP2 antibodies. Co-localization of EGF-Alexa Fluor 488 to LAMP-2-containing vesicles was examined. * *p < 0.05*. (D) *Pkd1* mutant (Null) MEK cells were treated, processed for immunostaining with anti-LAMP2 antibodies, and analyzed as in (C). * *p < 0.05*.

Deacetylation of α-tubulin by HDAC6 has been reported to destabilize microtubules and decrease motor binding.[19], [24] To determine whether microtubule stability affects EGFR degradation in renal epithelial cells, we treated *Pkd1* wild type and mutant MEK cells with nocodazole, which depolymerizes microtubules. Since ligand-induced degradation is the primary mechanism that controls EGFR levels, we also determined if microtubule stability affects EGFR degradation in response to EGF. *Pkd1* wild type and mutant MEK cells were treated with 0.2 µg/ml of nocodazole and/or 100 ng/ml of EGF. As expected, EGF alone induced the degradation of EGFR in *Pkd1* wild type and mutant MEK cells. However, EGF-induced EGFR degradation was strikingly attenuated in *Pkd1* wild type and mutant MEK cells treated with nocodazole for 2 hours (Fig. 8A and 8B). We further found that treatment with nocodazole strikingly decreased the transport of EGFR from early endosomes to late endosomes in *Pkd1* wild type and mutant MEK cells (*p < 0.05*) (Fig. 9A-9D). These results together with the finding that HDAC6 deficiency induces microtubule hyper-acetylation and promotes microtubule-mediated endocytic vesicle transport [16], [20] suggest that microtubule-mediated endocytic vesicle transport affects EGF induced EGFR degradation.

### Inhibition of HDAC Activity Decreased the Phosphorylation of ERK and Normalized EGFR Localization from Apical to Basolateral in *Pkd1* Knockout Mouse Kidneys

**Figure 10:**
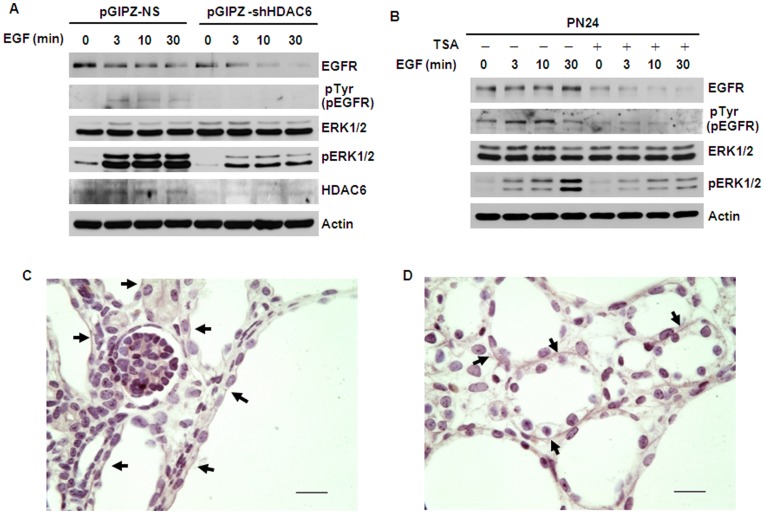
Inhibition of HDAC6 activity decreased the phosphorylation of ERK1/2 and normalized the localization of EGFR from apical to basolateral. (A) Knockdown HDAC6 with shRNA increases the degradation of EGFR and decreases the phosphorylation of ERK1/2. Serum-starved PN24 cells transduced with lentiviral plasmid pGIPZ-shHDAC6 or control empty vector pGIPZ-NS, were pretreated with 10 µg/ml cycloheximide for 2 hours and then stimulated with 100 ng/ml EGF for indicated times. (B) Inhibition of HDAC6 activity with TSA decreases the activation of EGFR and ERK1/2 . PN24 cells were pretreated with 100 ng/ml TSA for 12 hours and then starved for another 12 hours with TSA. After that, the cells were stimulated with 100 ng/ml EGF in the presence of 10 µg/ml cycloheximide and TSA for indicated times. (C) *and* (D) Treatment with TSA normalized the mislocalized EGFR from apical to basolateral in cystic epithelia in postnatal day 7 (P7) kidneys from *Pkd1^flox/flox^*:ksp-Cre pups. Immunohistochemical staining of EGFR in P7 kidney sections from *Pkd1^flox/flox^*:ksp-Cre neonates treated with DMSO (C) or TSA (D). Arrows pointed to apical (C) and basolateral (D) regions, respectively. Scale bar, 20 µm.

The EGFR axis is considered to act primarily through activation of the MAPK (RAS/RAF/MEK/ERK) pathway.[15] Since inhibition of HDAC6 activity increased the degradation of EGFR in renal epithelial cells, we suspected that it might also affect EGFR mediated ERK activation characterized with the levels of the phosphorylation of ERK1/2. To support this hypothesis, we found that knockdown HDAC6 with shRNA (Fig. 10A) or inhibition of HDAC6 activity with TSA (Fig. 10B) demonstrated a developmental decrease in EGFR and correlated with a substantial decreased phospho-ERK1/2 in cystic epithelial cells treated with EGF.

The mislocalization of the EGFR from basolateral to apical is known to occur in kidneys of both humans and mice and is a common end point associated with several different forms of polycystic kidney disease that are initiated by mutations in different genes.[7] We provided evidence that upregulated HDAC6 regulated EGFR endocytic trafficking and degradation in cystic epithelial cells. We suspected that upregulated HDAC6 might also contribute to the mislocalization of the apical EGFR on cyst lining epithelia. To test this hypothesis, we treated *Pkd1* ^flox/flox^:Ksp-cre pups with 0.5 µg/g TSA or DMSO (control) daily from postnatal day 3 (P3) to P6 and harvested the kidneys at postnatal day 7 (P7). Immunohistochemical staining of the control DMSO treated kidneys illustrates the expression of EGFR on apical cell surfaces of cystic epithelia (Fig. 10C). In contrast, treatment with TSA significantly increased the basolateral localization of EGFR on cystic epithelia (Fig. 10D), which suggested that inhibition of HDAC activity could normalize the mislocalized EGFR from apical to basolateral.

## Discussion

In this study, we provided the first evidence that 1) HDAC6 regulates EGFR intracellular trafficking and degradation in renal epithelial cells through HDAC6 mediated α-tubulin deacetylation and microtubule stability; 2) inhibition of HDAC activity normalizes apical EGFR to basolateral in *Pkd1* conditional knockout mouse model. We found that HDAC6 was up-regulated in *Pkd1* mutant MEK cells and in *Pkd1* hypomorphic homozygous (*Pkd1^nl/nl^*) kidney tissues (Fig. 1A–1D). Pharmacologic and shRNA mediated inhibition of HDAC6 decreased the levels of EGFR in renal epithelial cells (Fig. 2 and 3). We further found that HDAC6 regulated EGFR intracellular trafficking and degradation along microtubules in normal and mutant renal epithelial cells by mediating microtubule acetylation and stability since 1) inhibition of HDAC6 activity by pharmacological and shRNA mediated inhibition increased EGFR endocytic trafficking from early endosomes to later endosomes and increased the degradation of EGFR (Fig. 3 and 7); 2) treatment with nocodazole, which depolymerized microtubules, decreased the degradation of EGFR in *Pkd1* mutant MEK cells stimulated with EGF (Fig. 8) and EGFR endocytic trafficking from early endosomes to later endosomes and (Fig. 9); 3) pharmacological and shRNA mediated inhibition of HDAC6 also decreased the expression of phospho-ERK1/2, a EGFR axis mediated downstream target (Fig. 10A and 10B). In addition, inhibition of HDAC activity with TSA normalized the localization of EGFR from apical to basolateral of the cystic epithelial cells in *Pkd1* conditional knockout mouse kidneys (Fig. 10C and 10D). Thus, targeting HDAC6 to downregulate EGFR and its downstream targets as well as to normalize its localization from apical to basolateral may be a potential therapeutic approach for polycystic kidney disease.

Recent studies suggest that sorting and transport of early endocytic cargos to later stage endocytic compartments require microtubules, and HDAC6 may be involved in this process since HDAC6 destabilizes microtubules by deacetylating α-tubulin.[16], [19], [20] Inactivation of HDAC6 leads to dramatic stabilization and accumulation of acetylated microtubules.[24] Enhanced microtubule stability leads to the recruitment of molecular motors kinesin-1 and cytoplasmic dynein to microtubules thereby stimulating anterograde and retrograde vesicle transport including EGF stimulated EGFR.[17] The increased expression of HDAC6 and mislocalization of EGFR in cystic renal epithelial cells led us to explore the relationship between HDAC6 and EGFR in cystic epithelial cells. The data presented in this study demonstrate that HDAC6 contributes to increased expression of EGFR by disrupting microtubule mediated EGFR degradation in cystic epithelial cells. Our results show that inhibition of HDAC6 activity with TSA or HDAC6 shRNA significantly decreased the levels of EGFR and also decreased the EGFR axis mediated phosphorylation of ERK1/2 in *Pkd1* mutant renal epithelial cells. These data suggest that upregulation of HDAC6 may play a role in upregulating apical EGFR stability leading to prolonged EGFR signaling that facilitates cyst formation.

Previous reports from our laboratory demonstrated that inhibition of HDAC activity with TSA prevent renal cyst formation through targeting MEF2C or Id2 signaling, respectively.[25], [26] Our results that inhibition of HDAC activity with TSA could significantly normalize the mislocalized EGFR from apical to basolateral suggested a third possible mechanism for HDAC in mediating cyst development.

In sum, our study identified HDAC6 as a key component of the endocytic trafficking network in renal epithelial cells. The most exciting result related to translational application is that inhibition of HDAC6 activity with its specific inhibitor, tubacin, decreased the levels of EGFR and might also normalize the mislocalized EGFR in *Pkd1* mutant MEK cells. Thus, targeting HDAC6 to downregulate EGFR and also normalize EGFR localization may be a potential therapeutic approach to treat polycystic kidney disease.

## Concise Methods

### Cell Culture and Reagents

IMCD3 (murine inner medullary collecting duct) cells, HEK-293T cells were maintained at 37°C in 5% CO_2_ in DMEM (Invitrogen, CA) supplemented with 10% fetal bovine serum (FBS). Dolichos biflorus agglutinin (DBA) positive *Pkd1* wild type and *Pkd1* null cells mouse embryonic kidney (MEK) cells were maintained as previously described.[27] *Pkd1* heterozygous PH2 and homozygous PN24 cells were kindly provided by Dr. Stefan Somlo (Yale University).[28] In brief, *Pkd1* cell lines were derived from a single Pkd1^flox/−^ carrying a conditionally immortalizing, temperature and interferon-γ responsive transgene (H-2Kb-tsA58; ImmortoMouse, Charles River). Pkd1^−/−^ were produced from Pkd1^flox/−^ cells by transient transfection with a plasmid expressing the Cre recombinase. Passage numbers were kept below 20 for all cell lines. Cells were maintained in DMEM/F12 supplemented with 10% FBS and γ-interferon (5 U/ml; Sigma-Aldrich, MO) at 33°C and 5% CO2.[28] Trichostatin A (TSA), cycloheximide, nocodazole and saponin were purchased from Sigma-Adrich.

### Western Blot and Immunofluorescence

Western blotting was performed on whole-cell lysates as described by the manufacturer (Upstate Biotechnology, NY). The antibodies used for Western blotting and Immunofluorescence included anti-EGFR antibody (Cell Signaling technologies, MA; Santa Cruz, CA); anti-HDAC6 antibodies (Millipore, MA); anti-EEA1 (Santa Cruz, CA); anti-LAMP2 (Developmental Studies Hybridoma Bank); anti-acetylated-α-tubulin, anti-actin antibody (Sigma, MO). Donkey-anti-rabbit, donkey-anti-mouse, donkey-anti-goat IgG-horseradish peroxidase (Santa Cruz, CA) were used as secondary antibodies for Western blotting. EGF-Alexa Fluor 488 was purchased from Invitrogen (Invitrogen, CA). Fluro488-conjugated anti-mouse and anti-rabbit IgG antibody, Fluro555-conjugated anti-mouse, anti-rabbit, anti-goat, anti-rat IgG antibody (Invitrogen, CA) were used as secondary antibodies for immunofluorescence. Immunofluorescence images were obtained with a NIKON ECLIPSE 80i Microscope. For saponin treatment, cells were first rinsed with cold PBS three times and incubated with 0.005% (w/v) saponin that was made freshly in 4 °C PBS. Saponin treatment lasted 5 min on ice and was stopped by quickly rinsing the cells three times with cold PBS. Then regular immunostaining procedures were applied. For EGF treatment, cells were serum-starved overnight and then treated with EGF (100 ng/ml) or EGF-Alexa Fluor 488 (80 ng/ml).

### HDAC6 Knockdown by Lentivirus Carrying HDAC6 shRNA

HEK293T cells were cotransfected with lentiviral plasmid pGIPZ-siHDAC6 or control empty vector pGIPZ-NS, psPAX2 packaging plasmid, and pMD2.G envelope plasmid using calcium phosphate. Twelve hours later, medium containing the transfection reagent was removed and replaced with fresh complete DMEM medium plus 10% FBS and penicillin/streptomycin. Forty-eight hours later, cultures containing lentiviral particles were harvested from HEK293T cells. *Pkd1* wild type and mutant MEK cells and PN24 cells were then infected with appropriate amounts of lentiviral particles together with 5 µg/ml polybrene (Sigma, MO). Twenty-four hours later, virus-containing medium was removed and replaced with fresh medium plus 5 µg/ml puromycin. Two days after selection, all the cells are GFP positive, which indicates the efficiency of transduction. Five days after infection, cells were harvested and analyzed by Western blot to examine the efficiency of HDAC6 knockdown.

### Quantitative Reverse-transcription Polymerase Chain Reaction

HADC6 was stable knockdown in *Pkd1* mutant (Null) MEK cells. Cells were washed quickly with PBS, and total RNA was extracted using the RNeasy plus mini kit (Qiagen Germanton, MD). One micrograms of total RNA were used for reverse transcription reactions in a 20 µl reaction to synthesize cDNA using Iscript cDNA synthesis kit (BioRad, Hercules, CA). RNA expression profile was analyzed by real-time PCR using iTaq SYBER Green supermix with ROX (BioRad) and carried in an icycler iQTM Real-time PCR detection system. Genes were amplified using the following primers. EGFR-F: 5′-ATCAAAGTTCTGGGTTCGGG-3′; EGFR-R: 5′-GTTGGCTTTTGGAGATGTGG-3′; Actin-F 5′-AAGAGCTATGAGCTGCCTGA-3′; Actin-R: 5′-TACGGATGTCAACGTCACAC-3′. The complete reactions were subjected to the following program of thermal cycling: 40 cycles of 10s at 95°C and 20s at 63°C, a melting curve was run after the PCR cycles, followed by a cooling step. Each sample was run in triplicated in every experiment, and each experiment was repeated three times. The expression level of EGFR was normalized to the expression level of actin.

### Mouse Strain and Treatment

*Pkd1* ^nl^ mice were generated as described.[23] Homozygous *Pkd1* ^nl^ mice, with only 13–20% normally spliced *Pkd1* transcripts caused by an intronic neomycin-selectable marker, are viable, showing bilaterally enlarged polycystic kidneys. *Pkd1-floxed:*Ksp-cre mice were generated as described.[28] Each *Pkd1 ^flox/flox^*:Ksp-cre pup was injected with 0.5 µg/g TSA or DMSO (control) daily from postnatal day 3 (P3) to P6. All the kidneys were harvested at postnatal day 7 (P7) for further analysis.

### Immunohistochemistry

For HDAC6 staining, kidneys were fixed with 4% paraformaldehyde overnight. Polyclonal goat anti-HDAC6 antibody (1∶40, Santa Cruz, CA), a peroxidase conjugate secondary antibody (1∶200, Sigma, MO), and DAB (3,3′-diaminabenzidine tetrahydrochloride) substrate system were used. For EGFR staining, kidneys were fixed with 4% paraformaldehyde for 60 min at 4°C, then stepwise dehydrated in acetone, and embedded in resin blocks using a Immuno-Bed Kit (Polyscience Inc., Warrington, PA). 3 µM sections were then cut, and immunohistochemical staining of the EGFR was conducted using the rabbit polyclonal anti-EGFR antibody (Abcam, Cambridge, UK) (1∶50). Images were analyzed with a NIKON ECLIPSE 80i Microscope.

### HDAC6 Enzymatic Activity Assay

The measurement of HDAC6 activity from cell lysates of *Pkd1* wild type, heterozygous and homozygous renal epithelial cell lines was carried out with a commercially available assay kit (Cayman Chemical, Ann Arbor, MI) with modification. In brief, sub-confluent cells were washed twice and collected in PBS. Cells were solubilized with 20 mM Tris/HCl buffer (pH 7.5) containing 137 mM NaCl, 2 mM EDTA, 10% glycerol, 1%Triton x-100 and protease inhibitor cocktail (Roche Molecular Biochemicals, Indianapolis, IN). Cell lysates from different cell types were incubated on ice for 10 min followed by sonication, and then centrifuged at 16000 ***g*** for 15 min at 4°C. Same amount of cell lysates (2 mg) from different cell types were then incubated with anti-HDAC6 antibody pre-coupled beads, in that anti-HDAC6 antibody (Santa Cruz Biotech, SC-5258) was coupled to 20% protein G agarose beads (GE Healthcare Life Sciences) in PBS in the presence of 5 mg/ml bovine serum albumin (Sigma, MO) for 6 hours at 4°C, overnight at 4°C. The next day, immunocomplex beads were washed four times with lysis buffer and resuspended in 140 µl HDAC assay buffer. Each immunoprecipitant was incubated with 200 µM acetylated fluorogenic substrate in HDAC6 assay buffer at 37°C. After 30 min, the lysine developer was added, and the mixture was incubated for another 15 min at room temperature. The enzyme activity was monitored using a fluorimetric assay. Fluorescence was measured using a TECAN infinite M200 PRO microplate reader (Tecan, Research Triangle Park, NC) with excitation at 360 nm and emission at 460 nm.

### Statistics

Data are presented as mean ± Standard error. An unpaired 2-tailed student’s *t* test was used to determine the significant differences. A *P* value less than 0.05 is considered significant.

### Ethics Statement

This study was carried out in strict accordance with the recommendations in the Guide for the Care and Use of Laboratory Animals of the National Institutes of Health. The protocol was approved by the Institutional Animal Care and Use Committee (IACUC) of Medical College of Wisconsin (Permit Number: AUA1152).
